# Characteristics and phylogenetic analysis of the complete chloroplast genome of *Mesembryanthemum cordifolium* L. F. (Aizoaceae)

**DOI:** 10.1080/23802359.2024.2398180

**Published:** 2024-09-24

**Authors:** Wei Fu, Lin Li, Shuang Li, Yajie Li, Juzhi Sun, Liang Zhang, Yingchun Zou

**Affiliations:** Enshi Tujia and Miao Autonomous Prefecture Academy of Agricultural Sciences, Enshi, China

**Keywords:** *Mesembryanthemum cordifolium*, complete chloroplast genome, phylogenetic analysis

## Abstract

*Mesembryanthemum cordifolium*, a perennial plant with crassulacean acid metabolism (CAM) in the Aizoaceae family, has significant ornamental and medicinal values. In this study, we reported the first complete chloroplast genome sequence of this species. The total genome size was 153,734 bp in length, including a large single-copy (LSC) region of 85,692 bp, a small single-copy (SSC) region of 18,212 bp, and a pair of inverted repeat (IR) regions of 24,915 bp by each. The overall GC content of the *M. cordifolium* chloroplast genome was 37.08%. The genome encodes 131 genes, comprising 87 protein-coding genes (PCGs), 36 transfer RNA genes (tRNAs), and eight ribosomal RNA genes (rRNAs). Phylogenetic analysis shows this species was relatively close to *M. crystallinum*. This chloroplast genome sequence will be valuable for species discrimination and for understanding phylogenetic relationships within the genus *Mesembryanthemum*.

## 1. Introduction

Aizoaceae is the largest succulent family, with more than 2300 species, mainly located in tropical and subtropical climates, especially along the shore or in arid areas (El-Raouf 2021). *Mesembryanthemum cordifolium* L. F. 1782, also called *Aptenia cordifolia*, is a perennial plant with crassulacean acid metabolism (CAM) in the Aizoaceae family (Germishuizen and Meyer 2003; Gaffney 2006). Numerous studies have revealed that *M. cordifolium* contains alkaloids and has good resistance to abiotic stresses, such as salt (Cela and Munné-Bosch 2012) and drought (Cela et al. 2009). Additionally, *M. cordifolium* is not only utilized as a folk antidepressant remedy (Germishuizen and Meyer 2003; Said et al. 2021) but also planted in most parts of the world to enhance green spaces and gardens (Mohajjel Shoja et al. 2021). The chloroplast is a vital organelle in higher plants, serving as a metabolic hub for photosynthesis (Freudenthal et al. 2020). Due to its modest size and genome sequence reorganizations compared to the nuclear genome, the chloroplast genome has been extensively utilized in plant phylogenetic investigations (Javaid et al. 2022). With the innovation of next-generation sequencing technology, the chloroplast genomes of many plants have been completely sequenced and deposited on NCBI. However, there are only several complete chloroplast genomes of Aizoaceae species (*Sesuvium portulacastrum*, *M. crystallinum*, and *Tetragonia tetragonoides*) available on NCBI (Choi et al. 2018), which hampers a deeper understanding of genomic characterization and evolutionary history in the Aizoaceae family. Therefore, we reported the first complete chloroplast genome of *M. cordifolium* to enrich the genetic information of *M. cordifolium* and contribute to the species identification of the *Mesembryanthemum* genus.

## 2. Materials

**Figure 1.**
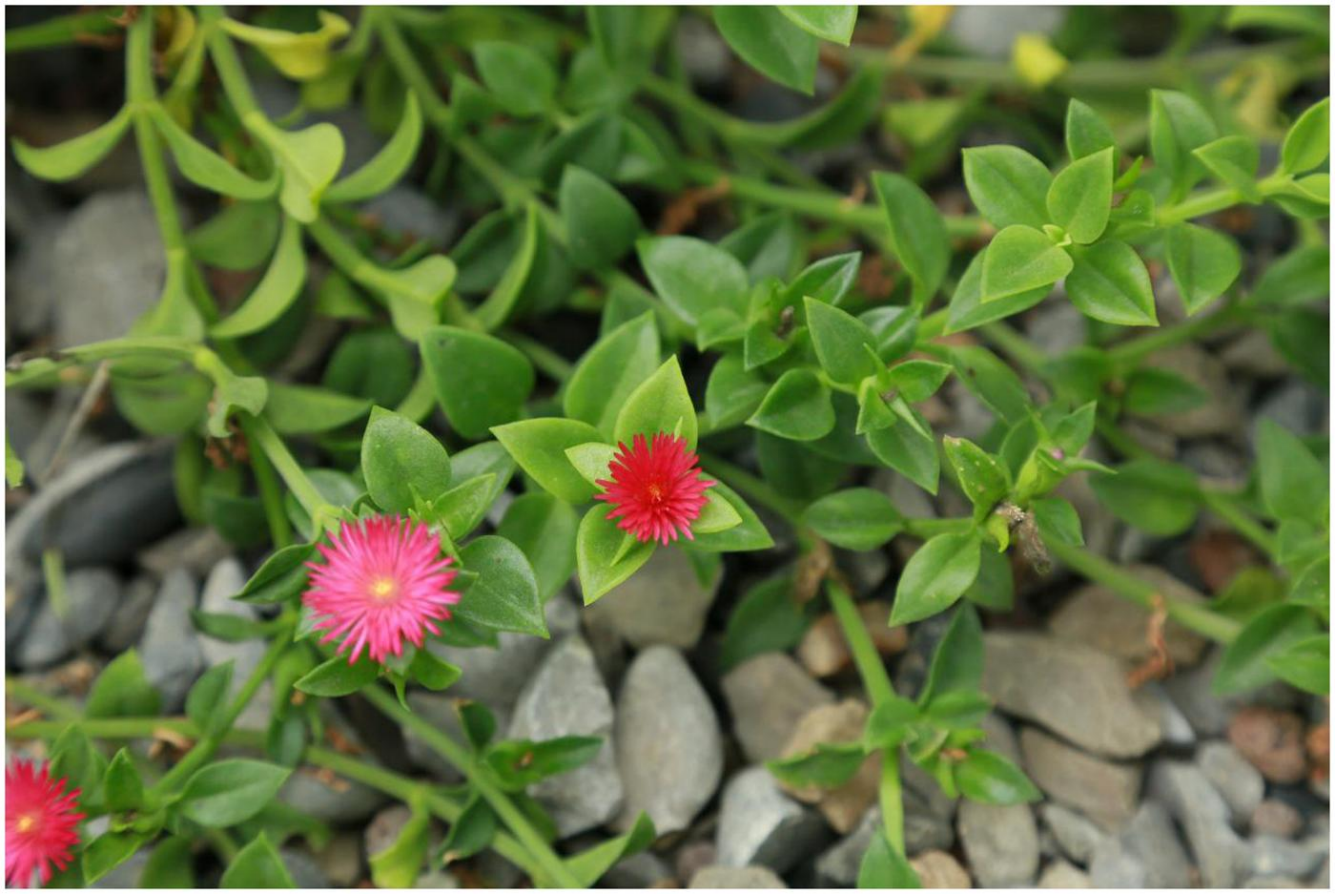
Species reference image of *M. cordifolium*. The photograph was taken by Lin Li at the germplasm resource nursery of Enshi Tujia and Miao Autonomous Prefecture Academy of Agricultural Sciences, Hubei Province, China, in September 2023.

Fresh samples of *M. cordifolium* were collected from the germplasm resource nursery of Enshi Tujia and Miao Autonomous Prefecture Academy of Agricultural Sciences, Hubei Province, China (N 30°31′53″, E 109°48′11″) (Figure 1). A specimen was deposited at the Herbarium of Enshi Tujia and Miao Autonomous Prefecture Academy of Agricultural Sciences (contact: Wei Fu, fuwei5@mail2.sysu.edu.cn) under the voucher number *FL-TQ20230901*.

## 3. Methods

Total genomic DNA was extracted from the fresh leaves using the modified cetyl trimethyl ammonium bromide (CTAB) method (Doyle and Doyle 1987) and then randomly sheared to yield approximately 350 bp fragments by sonication for library construction. After amplifying the DNA fragments with a conventional PCR reaction, we used the Agilent 5400 system (Agilent, Santa Clara, CA) to assess the quality of the library. Paired-end sequencing of whole sequences from both ends of 150 bp fragments was performed on the DNBSEQ-T7 platform (Beijing Biomics Tech Co., Ltd., Beijing, China). The raw reads were filtered to obtain high-quality reads by removing adapters, low-quality sequences (reads with unknown bases ‘N’), and reads with more than 50% low-quality bases (quality value ≤10) with the Trimmomatic software (Version 0.39) (Bolger et al. 2014). The clean reads were obtained to *de novo* assemble the chloroplast genome of *M. cordifolium* using GetOrganelle (version 1.7.1) (Jin et al. 2020) with the k-mer length: 21, 35, 45, 65, 85, and 105. With the published *Mesembryanthemum crystallinum* chloroplast genome (OR567248) annotation as the reference, the assembled chloroplast genome was annotated using CPGAVAS2 (Shi et al. 2019). CPGView (http://www.1kmpg.cn/cpgview) was used to improve annotation, visualize the structure of the chloroplast genome, and identify cis-splicing genes and trans-splicing genes (Liu et al. 2023). Additionally, simple sequence repeats (SSRs) in *M. cordifolium* were searched using the MISA Perl script (Thiel et al. 2003) with the following minimum number of repeats: 10 for mono, six for di-, and five for tri-, tetra-, penta-, and hexanucleotide SSRs. Finally, we submitted the annotated genomic sequence to GenBank with the accession number (OR804507).

To investigate the phylogenetic relationship of *M. cordifolium*, the chloroplast genome sequences of 28 species of the order Caryophyllales from 24 families and one outgroup plant were downloaded from the NCBI database. The PhyloSuite (Version 1.2.2) (Zhang et al. 2020) was used to extract protein-coding sequences from the chloroplast genome annotation files, while the MAFFT (Version 7.407) (Katoh and Standley 2013) was employed to align these protein-coding sequences. Then, the aligned sequences were concatenated using PhyloSuite (Version 1.2.2) (Zhang et al. 2020), and Modeltest (Version 3.7) was used to estimate the best-fit model of nucleotide substitution (Posada and Crandall 1998). With *Panax ginseng* (MH049735) (Wang et al. 2018) as an outgroup, a maximum-likelihood (ML) phylogenetic tree was constructed by IQtree (Version 1.7) (Nguyen et al. 2015).

## 4. Results

**Figure 2.**
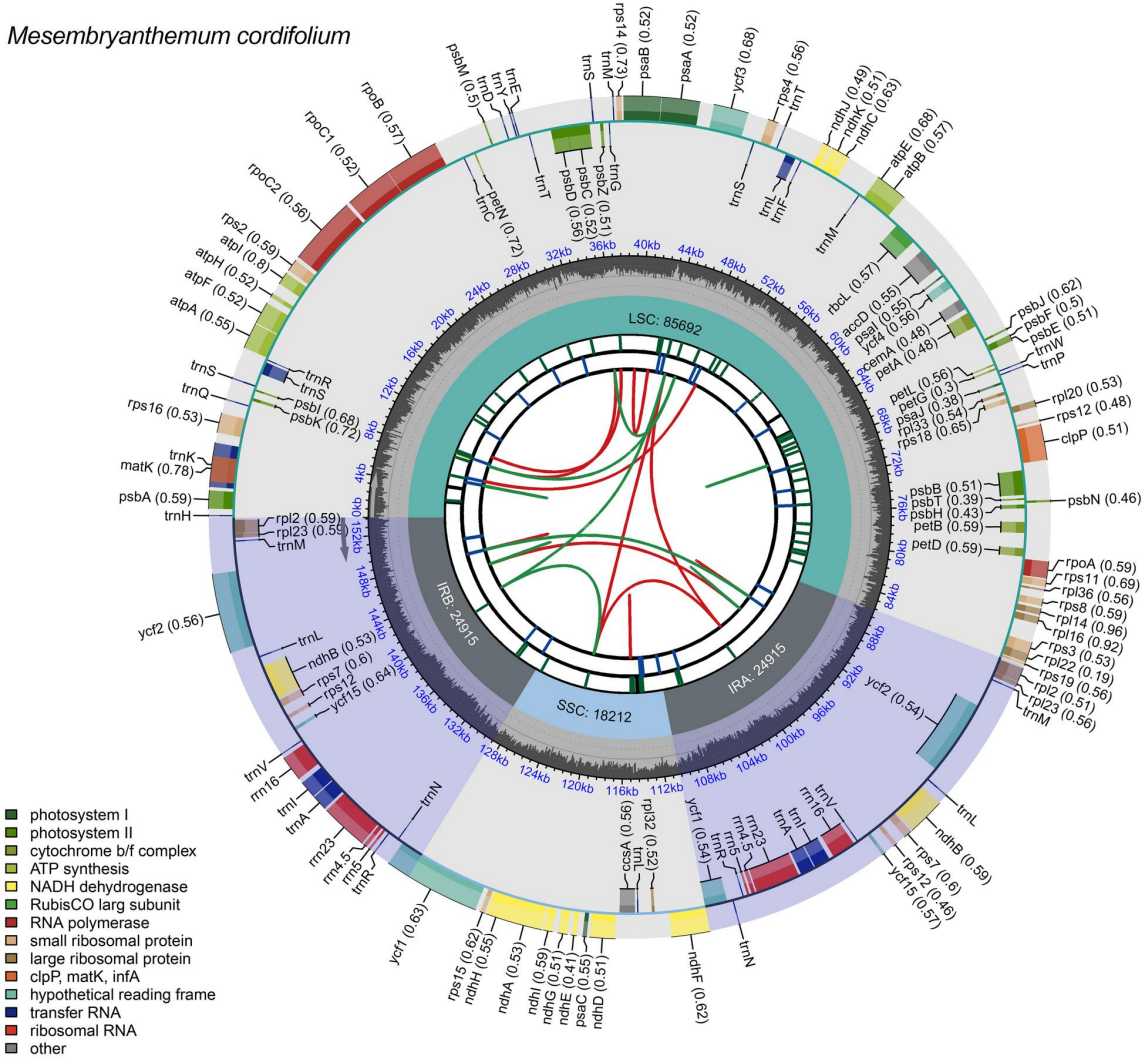
Genetic map of the chloroplast genome of *M. cordifolium*. Genes were classified by their function in colors. From the center outward, the first track shows the dispersed repeats. The dispersed repeats consist of direct (D) and palindromic (P) repeats, connected with red and green arcs. The second track shows the long tandem repeats as short blue bars. The third track shows the short tandem repeats or microsatellite sequences as short bars with different colors. The colors, the type of repeat they represent, and the description of the repeat types are as follows. Black: c (complex repeat); green: p1 (repeat unit size = 1); yellow: p2 (repeat unit size = 2); purple: p3 (repeat unit size = 3); blue: p4 (repeat unit size = 4); orange: p5 (repeat unit size = 5); red: p6 (repeat unit size = 6). The small single-copy (SSC), inverted repeat (IRa and IRb), and large single-copy (LSC) regions are shown on the fourth track. The GC content along the genome is plotted on the fifth track. The genes are shown on the sixth track. Genes are color-coded by their functional classification. The transcription directions for the inner and outer genes are clockwise and anticlockwise, respectively. The functional classification of the genes is shown in the bottom left corner.

In this study, we successfully obtained 35,843,960 paired-end reads, each with a sequence length of 150 bp, while subsequent filtering resulted in 35,827,328 clean reads. The complete chloroplast genome assembly of *M. cordifolium* was 153,734 bp in length, showing a typical quadripartite structure (Figure 2). There was a large single-copy (LSC, 85,692 bp) region, a small single-copy (SSC, 18,212 bp) region, and a pair of inverted repeat regions (IRs, 24,915 bp). The chloroplast genome GC content was 37.08%, with the LSC, SSC, and IR being 34.97%, 30.64%, and 43.05%, respectively. The average coverage depth of the *M. cordifolium* chloroplast genome is shown in Supplementary Figure 1. The chloroplast genome of *M. cordifolium* encoded 131 genes, including 87 protein-coding genes (PCGs), 36 transfer RNA genes (tRNAs), and eight ribosomal RNA genes (rRNAs). The introns were detected in 15 genes, with 13 genes (*atpF*, *ndhA*, *ndhB* (x2), *rpoC1*, *rps16*, *trnA-UGC* (x2), *trnI-GAU* (x2), *trnK-UUU*, *trnL-UAA*, and *trnS-CGA*) containing one intron and two genes (*ycf3* and *clpP*) containing two introns (Supplementary Table 1). Eight cis-splicing genes (*rps16*, *atpF*, *rpoC1*, *ycf3*, *clpP*, *ndhB(x2)*, and *ndhA*) and the trans-splicing gene, *rps12*, were detected by CPGView, and gene structures are shown in Supplementary Figure 2. We also identified 63 SSRs, including 62 mononucleotides and one dinucleotide (Supplementary Tables 2–4).

**Figure 3.**
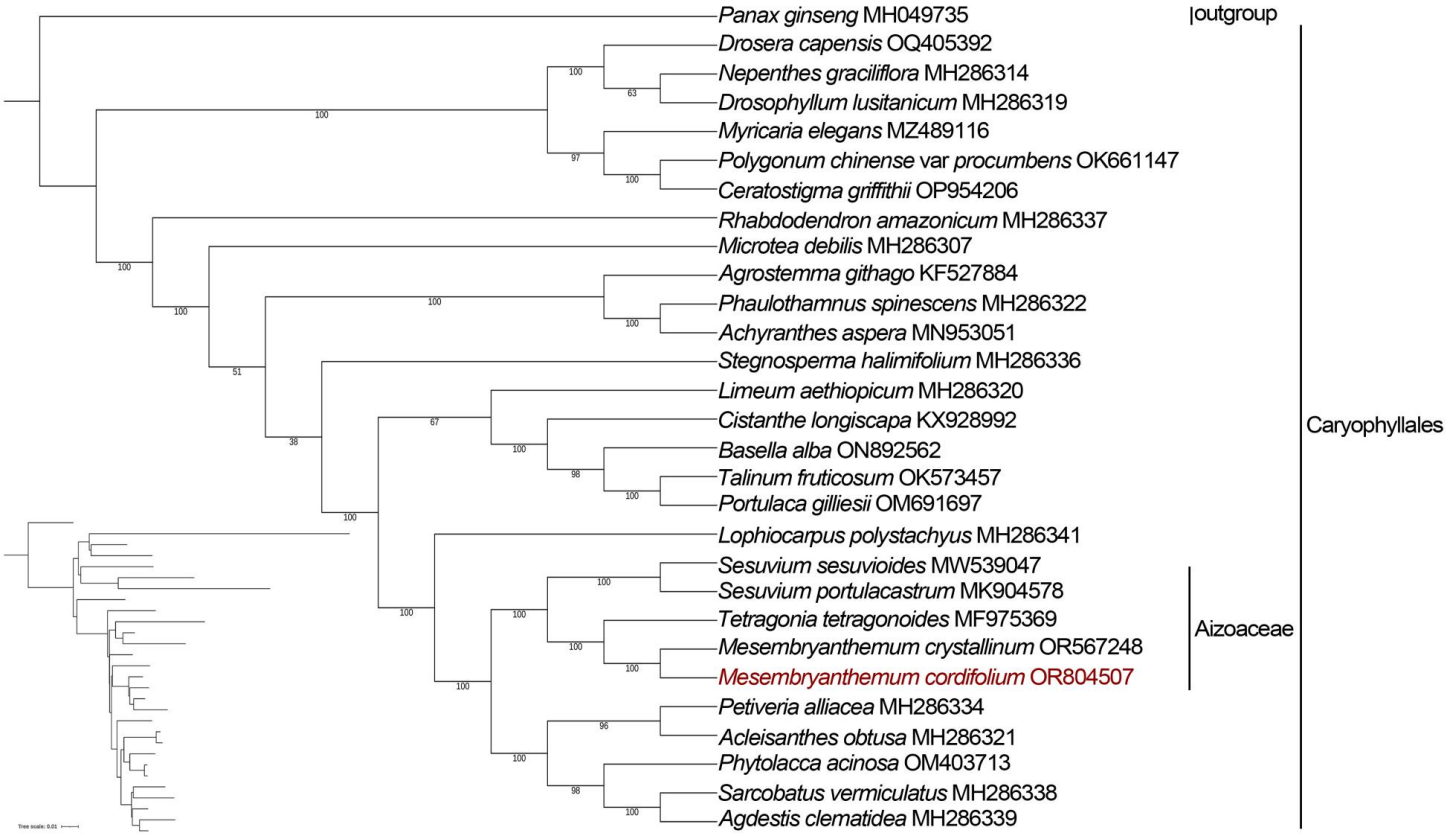
Phylogenetic relationships of *M. cordifolium* were inferred from maximum likelihood (ML) based on 29 chloroplast genomes, with *panax ginseng* as an outgroup. Ultrafast bootstrap values are shown below the nodes, with 1,000 bootstrap replicates. All the complete chloroplast genome sequences are available in GenBank. GenBank accession numbers: *panax ginseng* (MH049735) (Wang et al. 2018), *Drosera capensis* OQ405392, *Nepenthes graciliflora* MH286314 (Yao et al. 2019), *Drosophyllum lusitanicum* MH286319 (Yao et al. 2019), *Myricaria elegans* MZ489116 (Han et al. 2021), *Polygonum chinense* var. *procumbens* OK661147 (Zhang et al. 2022), *Ceratostigma griffithii* OP954206, *Rhabdodendron amazonicum* MH286337 (Yao et al. 2019), *Microtea debilis* MH286307 (Yao et al. 2019), *Agrostemma githago* KF527884 (Sloan et al. 2014), *Phaulothamnus spinescens* MH286322 (Yao et al. 2019), *Achyranthes aspera* MN953051 (Xu et al. 2020), *Stegnosperma halimifolium* MH286336 (Yao et al. 2019), *Limeum aethiopicum* MH286320 (Yao et al. 2019), *Cistanthe longiscapa* KX928992 (Stoll et al. 2017), *Basella Alba* ON892562, *Talinum fruticosum* OK573457, *Portulaca gilliesii* OM691697, *Lophiocarpus polystachyus* MH286341 (Yao et al. 2019), *Sesuvium sesuvioides* (MW539047) (Javaid et al. 2023), *Sesuvium portulacastrum* (MK904578), *Tetragonia tetragonoides* MF975369 (Choi et al. 2018), *Mesembryanthemum crystallinum* OR567248, *Petiveria alliacea* MH286334 (Yao et al. 2019), *Acleisanthes obtuse* MH286321 (Yao et al. 2019), *Phytolacca acinosa* OM403713, *Sarcobatus vermiculatus* MH286338 (Yao et al. 2019), and *Agdestis clematidea* MH286339 (Yao et al. 2019).

Based on 29 chloroplast genome sequences, including 28 species of the order Caryophyllales and one outgroup species (*Panax ginseng*: MH049735), the ML tree was constructed using IQtree with the best-fit model of GTR + F + I + G4. The phylogenetic analysis showed high bootstrap values for most of the nodes in the phylogenetic tree (Figure 3), and *M. cordifolium* was more closely related to *M. crystallinum*. The complete chloroplast genome of *M. cordifolium* described in this study can be further utilized for phylogenetic analysis, DNA barcoding, and molecular marker studies to differentiate at the species and variety level.

## 5. Discussion and conclusions

In this study, we obtained the complete chloroplast genome of *M. cordifolium* through high-throughput sequencing analysis and further analyzed the genomic structures, gene contents, and SSRs, which is consistent with the fundamental structural characteristics of angiosperm chloroplast genomes (Daniell et al. 2016; Yan et al. 2023). The phylogenetic tree results further revealed that *M. cordifolium* was more closely related to *M. crystallinum*, consistent with Yao et al. (2019). This chloroplast genome not only enriches the genomic information of *Mesembryanthemum* but also lays the foundation for understanding the evolution of Aizoaceae species. Our study provides a genetic resource for further genetic and genomic analyses of the genus *Mesembryanthemum*, which will be helpful for phylogeny, species identification, species resource conservation, and genetic investigations.

## Supplementary Material

Supplementary Figure.docx

Supplementary tables.xlsx

## Data Availability

The genome sequence data that support the findings of this study are openly available in GenBank of NCBI at https://www.ncbi.nlm.nih.gov/ under the accession no. OR804507. The associated BioProject, SRA, and Bio-Sample numbers are PRJNA1040120, SRR26826516, and SAMN38242416, respectively.
