# Supplementary material for: Characteristics and phylogenetic analysis of the complete chloroplast genome of Mesembryanthemum cordifolium L. F. (Aizoaceae)
Source: Mitochondrial DNA B Resour. 2024 Sep 24;9(9):1258–62. doi: 10.1080/23802359.2024.2398180 (PMC11423526; doi:10.1080/23802359.2024.2398180)
Supplement: Supplementary Figure.docx [file TMDN_A_2398180_SM3835.docx]

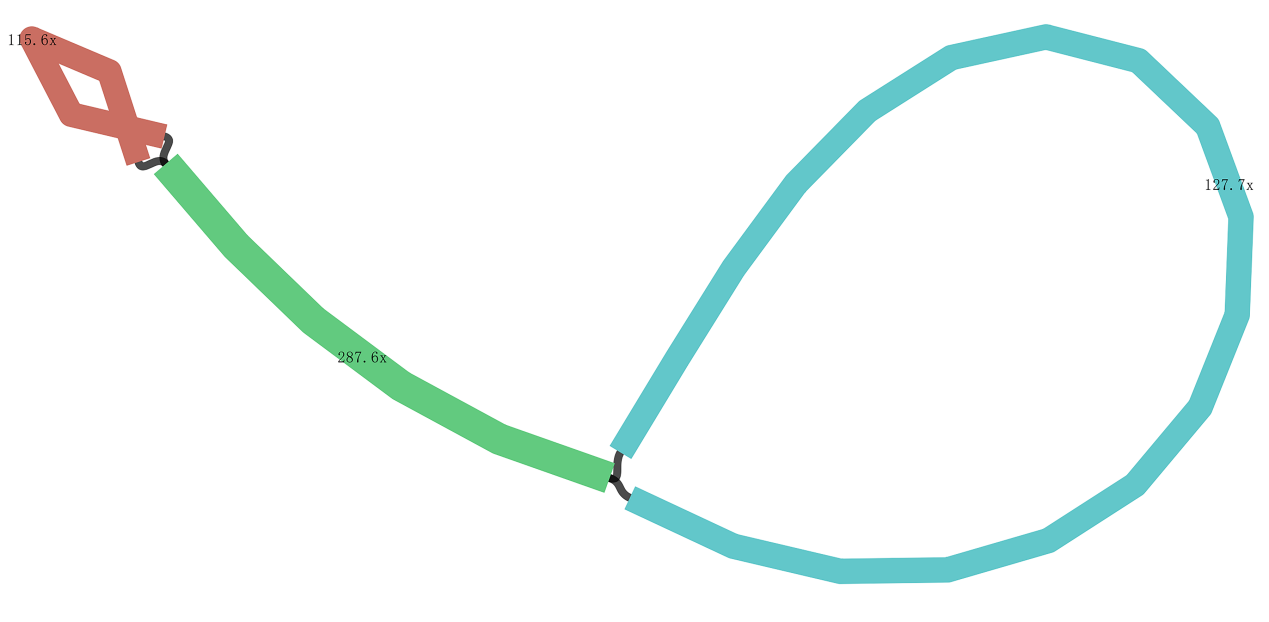


**Supplementary Figure 1**. Coverage depth distribution of the *M. cordifolium* chloroplast genome.


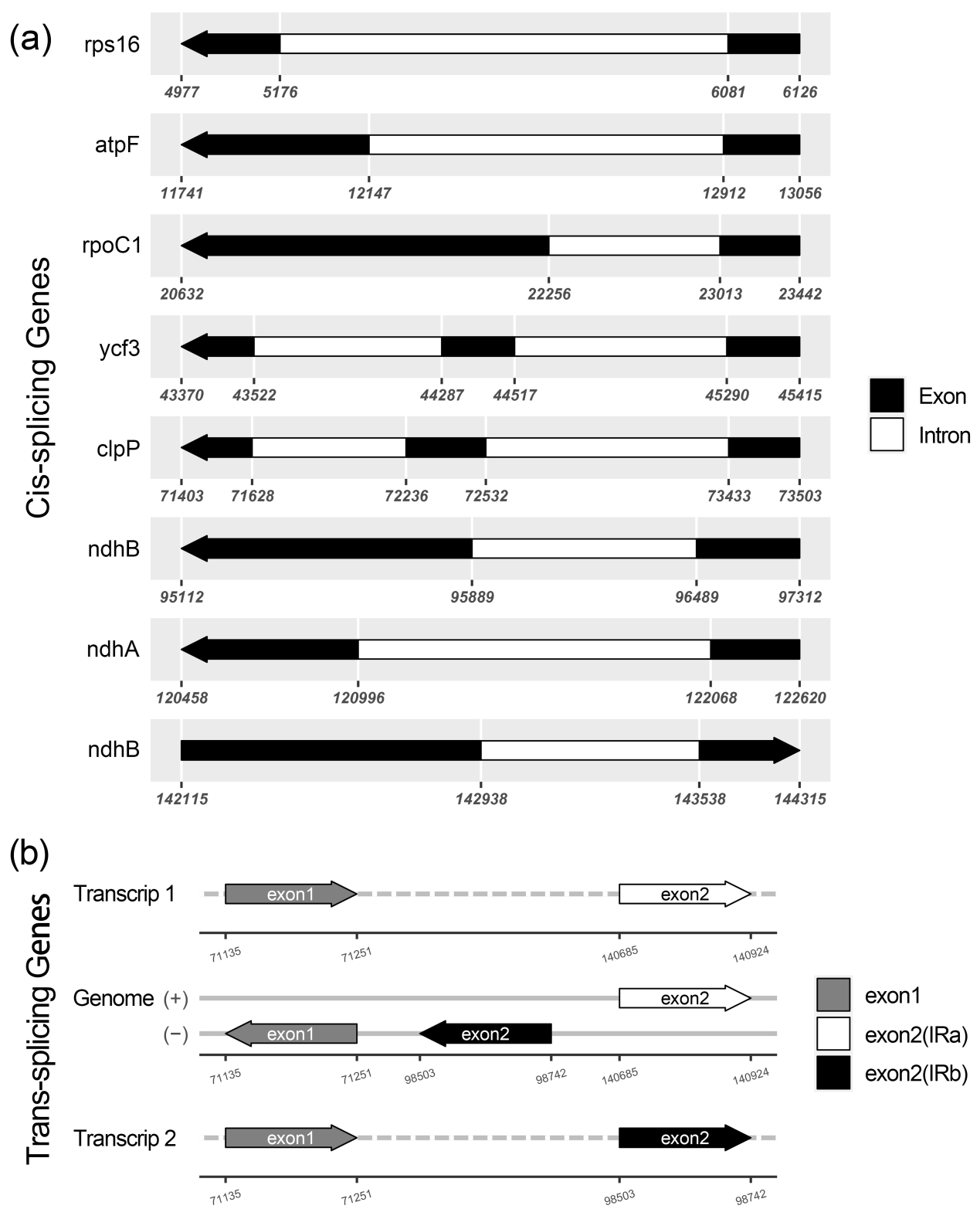


**Supplementary Figure 2**. Schematic of the cis-splicing gene map (a) and trans-splicing genes map (b) generated for the chloroplast genome of *M. cordifolium*.
